# Leveraging public awareness and behavioural change for entrepreneurial waste management

**DOI:** 10.1016/j.heliyon.2024.e40063

**Published:** 2024-11-01

**Authors:** Emma Etim

**Affiliations:** School of Geography, University of Nottingham, United Kingdom

**Keywords:** Behavioural change, Entrepreneurship, Freemium-to-premium, Municipal solid waste management, Public awareness, Theory of planned behaviour

## Abstract

This article takes us into the world of municipal solid waste entrepreneurs, revealing how public awareness campaigns and behavioural change efforts intertwine with entrepreneurial endeavours. Through conversations with 11 key participants—entrepreneurs from four geopolitical zones of Nigeria and a senior management figure from the Lagos State Waste Management Authority—I hear firsthand accounts of the challenges they face and their strategies to transform waste into opportunity. Guided by the theory of planned behaviour, this study sheds light on how these entrepreneurs perceive the role of public awareness in shaping waste management behaviour and driving business innovation. From their stories, a common theme emerges: the need to educate the public about responsible waste practices while developing creative business models that engage communities. Concepts like “freemium-to-premium” offerings are explored as ways to break through psychological barriers and inspire wider participation in waste management initiatives. These voices illustrate how awareness campaigns are not only about fostering environmental responsibility, but also about creating pathways for entrepreneurial growth, ultimately contributing to a more sustainable and circular economy. This study contends that public awareness extends beyond mere knowledge; it acts as a form of infrastructure in its own right. Rather than being just an end goal, awareness becomes a vital foundation in the pursuit of improved waste management practices, serving as a key driver for meaningful environmental action.

## Highlights

• Public awareness supports “ease of doing business”.
• Awareness goes beyond mere knowledge; it is a form of infrastructure in its own right.
• Awareness campaigns create pathways for waste entrepreneurial growth.
• Waste management education should be integrated into school curricula.
• “Freemium-to-premium” offerings are ways to break through psychological barriers.

## 1 Introduction

Amidst the cacophony of daily life and the infamous Lagos traffic jams, Rita, a municipal solid waste entrepreneur, encountered a situation one Tuesday evening that spurred a profound reflection on the state of waste management in her community. Immobilised by the gridlock, she observed a pedestrian on a congested road. The young man, while casually munching on biscuits and soda, nonchalantly discarded the empty can onto the street, despite a trash bin being just a few steps away. This act of littering, in the face of accessible waste disposal options, left Rita both perplexed and disheartened. Why, she wondered, would someone choose to litter when the means to dispose of waste properly were readily available?

Rita's encounter highlights the need to investigate the effectiveness of public awareness campaigns in promoting sustainable discard behaviour and practices. Municipal solid waste management (MSWM) remains a significant challenge for developing countries due to the lack of adequate regulatory, infrastructural, economic, and social instruments [[1], [2], [3], [4], [5], [6], [7]]. This study focuses on the social instruments, especially public awareness and behavioural change as tools to improve waste management entrepreneurship, while recognising the significance of other instruments such as infrastructure and regulations [8]. Nigeria, with a population of about 216 million generates approximately 25 million tons of municipal solid waste [9,10]. “*Waste generation has constantly increased in Nigeria due to increase in population”* [11], showcasing the need for more eco-entrepreneurs’ involvement and innovative waste management [12]. Public awareness enhances entrepreneurial activities by fostering community participation, encouraging proper waste disposal, supporting recycling initiatives, and promoting sustainable behavioural changes [[13], [14], [15]].

Entrepreneurs, such as Rita, involved in waste collection, recycling, and waste-to-energy (WtE) initiatives, benefit from increased awareness and an informed public that values sustainable waste practices and WtE technologies [16,17]. Moreover, increased public awareness, acceptance and participation leads to successful business, job creation and community development, as MSWM businesses require skilled workforce across its value chain [18,19]. Beyond economic advantages, promoting waste management is crucial for reducing pollution and conserving resources [20,21]. Effective MSWM yields significant public health and environmental benefits [13,[22], [23], [24]]. Therefore, increase public awareness of proper waste disposal and recycling will improve waste segregation and reduce landfill usage [13,23,25,26]. Studies have explored the role of public awareness in successful MSWM [12,14,27,28], the importance of entrepreneurial engagement [29,30] and how entrepreneurs are important change agents in waste management [31]. However, few studies have directly examined African entrepreneurs' perspectives on how public awareness impacts their waste management businesses and their role in promoting awareness within the ecosystem, which this study aims to address.

Understanding entrepreneurs' perspectives on public awareness can provide critical insights for developing more effective policies and campaigns to enhance sustainable waste management practices. This qualitative study applies Ajzen's theory of planned behaviour (TPB) [32] to explore how attitudes, subjective norms, and perceived behavioural control influence waste related behaviours. Specific objectives of this study include to assess entrepreneurs' perceptions of the significance of public awareness, evaluate entrepreneurs' contributions in enhancing public awareness, identify and analyse existing challenges, and explore entrepreneurs' perspectives on strategies to address these challenges. Findings from this study emphasise the importance of integrating public education with innovative business models, such as “*freemium-to-premium*” strategies, to overcome psychological barriers and enhance public engagement. These insights suggest that targeted awareness campaigns can not only foster environmental stewardship but also drive business growth by creating demand for waste management services. The remaining part of this article is divided into 5 sections: TPB and entrepreneurial engagement, methods, results and discussion, implications and limitations, and conclusion.

## 2 TPB and entrepreneurial engagement

TPB, developed by Icek Ajzen, offers a lens through which individual behaviours can be both understood and shaped [31]. Its relevance extends into the domain of recycling, where it serves as a framework for enhancing waste management practices [33]. This framework is structured around three core elements: attitudes, subjective norms, and perceived behavioural control, all of which influence behavioural intentions [34]. When we turn to waste management, we find that fostering positive attitudes is often a matter of education and continuous awareness creation. It's through campaigns that emphasise benefits and showcase successful global practices that the community can come to align with the larger movement towards sustainable waste attitudes and practices such as reducing, reusing and recycling [35]. But attitudes alone aren't sufficient. Community engagement becomes vital here, where the norms of the group—how people see their neighbours, friends, and fellow citizens engaging with waste—shape collective action [36]. Within these social norms lies the individual, with their own intentions to act. These intentions, however, need the right conditions to transform into habitual behaviours.

For intentions to materialise into everyday practices, infrastructure needs to improve, educational resources must be provided, and practical barriers addressed. One such barrier is the widespread failure to view waste as a resource. People continue to treat waste as something merely to be discarded, ignoring its potential value. This behavioural intention is driven by lack of environmental consciousness [37]. This is where the role of entrepreneurs enters the narrative. Entrepreneurs see waste not as a burden, but as an opportunity—both environmentally and economically. Yet, public attitudes and views towards waste shape their decisions [38], even as entrepreneurs see profitable potential in what others discard. By highlighting business models that illustrate not just environmental sustainability but also profitability, attitudes towards waste entrepreneurship can shift, attracting both current and prospective entrepreneurs [39,40]. Moreover, educational initiatives that tie waste management practices to broader concerns like public health and urban cleanliness can further solidify the business case for sustainable waste management [13]. These efforts are buttressed by social norms that promote responsible waste handling [[41], [42], [43]]. Overall, while public awareness initiatives, including educational campaigns, serve as key drivers for entrepreneurial success in the MSWM sector, entrepreneurs themselves play a crucial role in shaping and strengthening public attitudes, social norms, and perceived behavioural control surrounding waste management.

## 3 Methods

This study embarks on a qualitative journey, guided by the TPB, to delve into the perspectives of entrepreneurs like Rita regarding the efficacy of public awareness campaigns in fostering sustainable MSWM behaviours. Nigeria, with its vast and diverse population, stands as a microcosm of urbanisation challenges—where infrastructural gaps, social complexities, and regulatory constraints converge, highlighting the urgent need for innovative waste management solutions that entrepreneurs are uniquely positioned to offer [44,45]. Qualitative methods are employed to capture the dynamic factors that shape these entrepreneurs' perceptions and actions [46,47]. This study unfolds through a series of interviews with entrepreneurs, carefully selected from four geopolitical zones across Nigeria, including a senior representative from the Lagos State Waste Management Authority (LAWMA), an organisation serving the municipal solid waste needs of over 21 million residents [48]. The study draws on the insights of 11 participants, encompassing seven semi-structured in-depth interviews and six checklist-based discussions (Appendix 1), balancing thematic consistency with the flexibility needed to explore the depth of each participant's experiences [49]. All interviews were conducted virtually in English. Semi-structured interviews were chosen as the most appropriate method for capturing the nuanced perspectives of entrepreneurs, a choice supported by recent studies [50]. With participants' consent, all interviews were recorded and subsequently transcribed. The transcripts, along with checklists, were analysed using NVivo 14, uncovering key themes, compelling quotations, and recurring keywords that provided deeper insight into the entrepreneurs' experiences and views [51] (Table 1). These elements were then interpreted within the context of the study's objectives, theoretical framework, and the broader body of literature, offering a rich tapestry of insights into the entrepreneurs' views on public awareness of MSWM and generating actionable recommendations for improvement.

**Table 1:** Research objectives, themes and selection of quotations.

Objectives	Themes	Selection of quotations	Keywords	Code
Entrepreneur's perception of the significance of public awareness of MSWM in Nigeria	Economic opportunities	*"Our model starts with freemium then at the end it becomes premium"*	Freemium to Premium Model	MS3a
		*"The more the awareness, the better and the ease of us doing our business"*	Ease of doing business	MS3b
	Environmental Impact	*"Help the environment alone"*	Environment	MS3c
		*"Effect its absence on their environment"*		TN2
	Social and health benefits	*"Effect this is having on especially our health"*	Health	KM4a
Entrepreneurs' perception of the challenges of MSWM in Nigeria	Facilities	*“What we did again was to get a polythene bag for most households”*	Households	CO5a
	Education and Training	*"This education gap, we are still having issues with it"*	Education	MS3d
		*"Create [educate] more experts into the industry"*		MS3e
	Monetary Value and Incentives	*"They will have to now attach monetary value to it"*	Monetary value	MS3f
		*"Some of them that are willing to do it expect high returns from it"*	High returns	CN1a
		*"They still get that same original value"*	Value	CN1b
	Cultural Attitudes	*"Our people have overtime developed a certain way of life, which I'll call a throw away culture"*	Throw away culture	CN1c
		*"In this part of the country that I am in the north, they believe that whatever happens to you is the will of God"*	Will of God	CO5b
	Awareness campaigns	*"In 2022, the Coca Cola foundation sponsored a two-month radio drama series"*	Radio drama series	CO5c
		*"When we came on board, we were able to reduce the way people dump refuse"*	Dump patterns	CO5d
		*"They are trying to put up billboards"*	Billboards	AA7
		*“This community engagement is more like having town hall meetings”*	Community engagement	MS3g
Entrepreneurs' perspectives on the strategies to curb MSWM challenges in Nigeria	Circular economy model	*“So, we are also starting our circular economy, but not on the full scale”*	Circular economy	LM6
	Innovative technologies	*"This community engagement is more like having town hall meetings"*	Community engagement and awareness	MS3h
	Research and development	*"Our R&D that is really working"*	R&D	MS3i
	Public-private partnerships	*"The government needs to collaborate with us"*	Collaboration	KM4b
	Regulatory and institutional support	*"There are no enforcement agencies"*	Enforcement	KM4c
		*If only the government could enforce because there are no enforcement agencies"*		

The validity of this study was anchored on two critical approaches. First, the data interpretations were drawn not from individual entrepreneurs as isolated voices, but from their collective perspectives as key actors within the waste management sector, offering insights shaped by their professional roles. This is even though this study included individual quotes which is a traditional way to support claims in the results and discussion section of qualitative studies. By considering entrepreneurs as key players within the waste management sector, their insights were understood in the context of their professional roles and experiences. This approach emphasises the broader, shared perspectives of these entrepreneurs within the industry, rather than focusing solely on individual opinions. Second, the research sample was thoughtfully divided into two distinct groups—waste management authorities and entrepreneurs. This division was not just a methodological choice but a deliberate strategy to reduce bias, allowing for a more robust and credible set of findings. Leung argued that the validity of a qualitative study lies in the methodological appropriateness and the robustness of analysis, which have all been captured in this study [52]. The reliability of the data was further ensured using a recording table, a tool that facilitated swift and accurate interpretation, capturing the essence of the narratives shared by those deeply embedded in the waste management landscape. In conducting this research, the ethical dimensions were rigorously upheld. Informed consent was obtained from all participants, ensuring that their confidentiality and anonymity were preserved throughout the study. The research was conducted under the ethical oversight of the Faculty of Social Science Research Ethics Committee at the University of Nottingham, Malaysia (FASS2023_0012/PHIR/EEE20546675).

## 4 Results and discussion

Rita's experience is a commonly overlooked dimension of the issue - public awareness, behaviour and participation. Her experience, like many others, speaks to the heart of how deeply intertwined these factors are with waste management in Nigeria. In this section, we listen to the voices of entrepreneurs as they reflect on the role of awareness campaigns in addressing municipal solid waste challenges and their own efforts to contribute to this cause (Table 1). These entrepreneurs are not only participants but key actors in raising public consciousness about waste management. A table detailing their specific contributions in promoting waste management awareness in Nigeria can be found in the supplementary materials, offering a closer look at their impactful efforts.

### 4.1 Entrepreneur's perception of the significance of public awareness of MSWM

For entrepreneurs like Rita, investing in public awareness is both a moral calling and a practical business strategy. Here, educating the public isn't just about doing the right thing—it's about creating a more efficient waste management system where everyone plays their part. When communities are informed and conscious about waste, the entire process runs smoother, and costly issues like littering and illegal dumping can be minimised. From the conversations with entrepreneurs, their perceptions of public awareness around MSWM naturally fall into three key areas: economic opportunities, environmental impacts, and social and health benefits. These categories reflect not just their business goals, but their commitment to making a broader difference in the communities they serve.

#### 4.1.1 Economic opportunities

As communities grow more aware of the significance of proper waste management, there is a noticeable shift—demand for waste-related services has surged. This heightened awareness pushes both individuals and businesses to prioritise efficient resource use and seek sustainable solutions [53,54]. Entrepreneurs like Rita have felt the ripple effect of this change. In response, she and others have expanded their operations, hiring more skilled workers to keep pace with the rising demand for services like waste collection, recycling, and WtE projects [55,56]. This expansion is not just a business decision, but a reflection of how deeply community consciousness can transform the waste management landscape. *“In honesty, the more awareness, the better, and the ease of doing our business”* [MS3b]. Businesses that effectively promote MSWM practices set themselves apart in the market, drawing eco-conscious customers and potentially increasing their market share. Vieira et al. emphasised the need to understand environmental attitudes and actions, while identifying psychological barriers—such as resistance to change, conflicting goals, and interpersonal dynamics—as significant challenges to behavioural change [57]. By offering initial services at no cost and providing thorough education on waste management, these strategies help overcome psychological barriers, lower entry barriers for customers, and gradually integrate monetary value, making the approach economically viable for both consumers and businesses. *“Our model starts with freemium; in the end, it becomes a premium after a few months [MS3a]. Therefore, within the freemium stages, we have to convince them, train them on things that they need to do, and tell them how it works and how they can key to it. Then, before the premium, they must attach a monetary value [MS3f]. Therefore, if there is heightened awareness within this space or the industry at large, believe that it is not just going to help the environment alone [MS3c], but even our business, it will definitely help us financially.”* – [MS3]. Townsend and Ackerman discuss the potential challenges of market saturation and competition [58]. Although intense competition and heightened public awareness cause profitability issues, differentiation through effective MSWM practices can address these issues, as entrepreneurs moving from freemium to premium can attract loyal customers, gain a competitive edge, and mitigate market saturation risks.

#### 4.1.2 Environmental impact

The data gathered in this study stresses a familiar yet powerful truth – public education about proper waste disposal and recycling is key to curbing pollution, reducing landfill dependency, and conserving vital resources [59]. However, as Teixeira and Guerra remind us, awareness alone is not enough. Challenges like inadequate infrastructure, limited resources, and rapid population growth must also be confronted [60]. These obstacles, acknowledged in the study's introduction, were categorised into regulatory, infrastructural, economic, and social deficits. Entrepreneurs, in this context, emerge as pivotal actors, stepping in to bridge the gap between awareness and meaningful environmental action. They provide the innovative solutions and infrastructure necessary for an effective waste management system, turning awareness into tangible change. *“A good number of them [members of the public] now understand the importance of MSWM, and more people are getting to understand its importance and the effect its absence [has] on their environment and consequences on the human as well”* [TN2]. Increasingly, individuals are becoming conscious of the profound impact that proper waste management has on their environment and the severe consequences of neglecting it. Ferronato and Torretta's earlier work has painted a vivid picture of the environmental toll that waste mismanagement can take—polluting the air, soil, and water [61]. In this context, growing public awareness becomes more than just knowledge; it functions as a form of infrastructure in its own right. This heightened consciousness fosters a deeper, more collective understanding of how poorly managed waste can harm both the environment and the health of the communities living within it [62,63]. Awareness, then, is not merely an outcome but a critical component in the fight for better waste management practices.

#### 4.1.3 Social and health benefits

Gaining public support for entrepreneurial innovations in waste management becomes much more achievable when people understand the direct impact that improper waste practices have on their daily lives. Clear communication about the social and health consequences—like the fact that mismanaged waste can create breeding grounds for pathogens and lead to the spread of diseases such as cholera, typhoid, and dysentery—resonates deeply. Knowing that contaminated air, water, and soil pose serious threats to community health helps people see waste management not as a distant issue, but as a pressing and personal concern. It is this awareness that fosters a stronger, more engaged public response to entrepreneurial efforts aimed at solving these challenges [[64], [65], [66], [67]]. Beyond the efforts of entrepreneurs to raise awareness through their services and campaigns, community engagement emerges as a critical component. A population burdened by poor health strains the community's healthcare infrastructure, whereas active participation in waste management initiatives encourages shared responsibility and involvement in waste reduction, segregation, and recycling. Community authorities can bolster these efforts by providing essential waste management tools, such as bins and personal protective gear, to both households and entrepreneurs. *“We can do this only at the local level and with the support of the authorities. Create awareness and educate community dwellers on the effect this has on our health and environment.” – [KM4a]. “… So, yes, I can say we are rising from that point where nobody knew about it before to the point where we can say yes, we know one or two things because we are even acting on it.” – [MS3].* A previous study agreed that community engagement can enhance social cohesion and create a shared commitment to maintaining a clean and healthy environment, which benefits everyone involved [68].

### 4.2 Contributions of entrepreneurs in promoting MSWM awareness

The activities of entrepreneurs in waste management can be classified into high, moderate, balanced, and low levels of engagement, reflecting the diverse priorities shaped by practical constraints such as limited investment, human resources, and geographic challenges [13,69]. High engagement efforts, embraced by all the entrepreneurs, centre around collaborating with local community leaders to spread awareness about waste management. This culturally attuned approach, which proved effective during the COVID-19 pandemic, taps into the strong influence of community leaders to shape waste management behaviours [[70], [71], [72]]. Entrepreneurs emphasise that using similar strategies—such as billboards, radio, TV, flyers, jingles, door-to-door outreach, SMS, and social media—could further elevate public understanding of waste management. Moderate engagement includes school campaigns, though financial limitations make it difficult to appoint dedicated awareness officers or maintain regular outreach efforts. Low engagement is seen in more resource-intensive methods, like printed materials, local radio broadcasts, mobile apps, SMS services, and toll-free helplines, where high costs and logistical hurdles constrain widespread use [73,74].

The limited infrastructure and technological penetration, along with low literacy and mistrust in new technologies, hinder the effectiveness of these initiatives [75,76]. As a result, entrepreneurs often rely on more direct and less resource-intensive methods. To enhance MSWM awareness, leveraging social media and local influencers could boost community engagement, while digital tracking and penalties could streamline enforcement. Collaborating with educational institutions and NGOs offers cost-effective campaigns, and digital platforms can ensure broader outreach [77]. Moving towards digital information hubs, engaging online content, and data analytics could modernise these efforts. Media companies (both traditional and social) and digital marketers should support these initiatives for greater impact [78,79]. Also, introducing mobile apps and SMS updates, along with toll-free helplines, could revolutionise information dissemination. Here, collaboration among mobile operators, app developers, and local authorities is vital for promoting accessible and user-friendly digital solutions [80].

### 4.3 Entrepreneurs’ perception of the challenges of MSWM

Entrepreneurs consistently point to a range of challenges in waste management, including a widespread lack of awareness, educational gaps, behavioural barriers, cultural and social norms, media influence, and difficulties in collaboration [81]. Despite government and NGO efforts to raise awareness, large segments of the population remain either uninformed or indifferent. Entrepreneurs observed that the public's grasp of MSWM complexities—such as waste segregation, recycling processes, and the environmental consequences of improper disposal—is still quite limited [82,83]. Resistance to new technologies and waste management systems is common, highlighting the need for greater investment in advocacy efforts to deepen public understanding and foster acceptance of more sustainable practices. *“When we came on board, we were able to reduce the way people dump to some extent [CO5d]. What we did again was to get a polythene bag for most households [CO5a], give it to them, and inform them that on a weekly basis, we will come and pick it up”* [CO5]. The gaps in education and information contribute significantly to this awareness deficit. Entrepreneurs stress the need for more public education on sustainable waste management and the importance of reducing, reusing, and recycling waste. Although organisations like United Nations Environment Programme (UNEP) offer training programs, they fall short of bridging this knowledge gap. Entrepreneurs entering the sector without education and training often lack the necessary technical skills and understanding of best practices, which hampers innovation and exacerbates the country's waste management challenges.

… this education gap, we still have issues with it. [MS3d]. For example, if anybody wants to enter waste management professionally in Nigeria, I do not know which school a person is going to attend. I do not even know the course you are going to take. Environmental space, if not for the United Nations. If not that they are trying to roll out courses through the UNEP. If we have a body that includes the educational aspect, we can create more experts in the industry [MS3e].

The absence of structured education and training in waste management has led to widespread ignorance among households regarding proper waste segregation and disposal methods. Occasional training from international bodies fails to meet the broader population's needs, resulting in poor public waste management behaviour and re-emphasising the need for higher educational institutions to contribute [84]. This knowledge gap fosters practices such as open dumping, littering, and waste burning, which harm the environment and pose significant health risks [85]. Even with extensive education on MSWM, behavioural challenges can hinder the application of knowledge. Issues include improper sorting of recyclables, reluctance to engage in composting programs, and general apathy towards waste management initiatives, often influenced by cultural and social norms. This aligns with the theory of planned behaviour, which explains that attitudes towards waste management are shaped by varying cultural and social norms.

… Our people have developed a certain way of life over time, which I will call a throw away culture [CN1c]. They don't care. They simply dispose of it. They simply throw this away. Now, when you tell them, this can be useful. This thing has some value: we need you to keep them, and we will come pick them up. Some expect high returns [CN1a]. Some expect more returns than the original value of the product. The PET value is 10 Naira. Now, some people expect that when you want to pick it as waste for recycling, they will still have the same original value [CN1b]. Sometimes you see someone who will say okay; instead of giving you at this amount, it is better to burn it, and they will go ahead and burn it. They will not give this to you. – [CN1].

Entrepreneurs recognise that in some communities, there is a strong culture of waste reduction and recycling, whereas in others, waste management is not prioritised or is seen as the responsibility of the government rather than individuals. Religious beliefs are also relevant. *“In this part of the country, where I am in the North, they believe that whatever happens to you is the will of God [CO5b]. Therefore, they do not take care of themselves as they are supposed to do, which is very bad. [Here], the government and religious leaders need to do more work. The people respect their religious leaders. So, whatever they [religious leaders] tell them, they follow suit. The government needs to let them know the implications of not keeping the environment clean.”* – [CO5]. Entrepreneurs have proposed the need for active media and communication of best practices in MSWM. The role of media and communication is crucial in shaping public perception [86]. Respondents acknowledged that, while there are efforts to highlight waste management issues in the media, more consistent and widespread coverage is needed to keep the issue at the forefront of public consciousness.

Another thing is that we also tried to appear in these events. I know my colleague; he is very good at appearing on radios, televisions, and even hosting webinars, right? We appear and talk to the people. Yes, even if that does not directly impact on our primary target audience, we also know that it is really helping the company's awareness and people know what we are in this space to do. - [MS3].

In 2022, the Coca-Cola Foundation sponsored a two-month radio drama series in Abia State because we are participating in the program [CO5c]. Therefore, within the two-month radio drama series, it was aired every Wednesday for 30 minutes. It was done in a bar, so once the program started at the end, my number would be shared on air. From that Wednesday to maybe Sunday, I will receive many calls from individuals trying to learn more about recycling and waste management and how they can benefit from the value chain. Through that drama series, the quantity of waste recovered from Abia State increased, and I think that if that drama series had not been completed, most of the waste would have ended where it would have been difficult for someone to recover. They might end up in drainage systems, rivers, streams, or even landfills. - [CO5].

Entrepreneurs also suggest leveraging social media and other digital platforms such as billboards. *“Therefore, they attempted to put up billboards. They are trying to put up posters and they do radio advertisements to create awareness, but I do not know how far that has been assimilated by the people, but I think with time the message they are trying to pass will get to more people.”* – [AA7]. The recognition of the importance of collaborative efforts between different stakeholders and government agencies, non-profits, businesses, and community groups—is essential to raising public awareness and driving change. Entrepreneurs advocated for more partnerships and community-based initiatives to educate and engage the public [87]. They also advocated for more community events targeted at enlightening people about MSWM best practices. *“Thus, the first is just like this community engagement, and this community engagement is more like having town hall meetings and organising events within their communities [MS3g]. Therefore, like the community we currently work with, every last Saturday of the month, we always have cleanup within the community. So, we make it a community engagement; they have to come, we talk to them, we tell them just more about business and how they can key in.”* – [MS3]. Through collaboration, the impact of awareness campaigns was amplified, and a culture of responsible waste management was fostered.

### 4.4 Entrepreneurs’ perspectives on the strategies to curb MSWM challenges

The incident in Lagos served as a catalyst for rethinking waste-management approaches. Rita's reflections highlight the need for a holistic strategy that integrates infrastructure development with robust public education initiatives. Entrepreneurs in the waste management sector must advocate for and participate in awareness campaigns, leveraging various platforms such as the circular economy model, enhanced public-private partnerships, and increased community engagement.

#### 4.4.1 Circular economy model

Respondents agreed that the transition from a linear to circular economy was crucial for addressing the mounting challenges of MSWM. In a linear economy, products are manufactured, used, and discarded, leading to significant waste and environmental degradation [88,89]. Conversely, a circular economy emphasises reducing waste, reusing materials, and recycling products to create a closed-loop system [90]. This model not only helps manage waste more effectively but also promotes sustainable economic growth by creating new business opportunities in the recycling and refurbishing sectors. *“When we look at the international space, we see that they have gone beyond collection and disposal or collection and reuse to a circular economy. Therefore, we are also starting our circular economy, but not at full scale.”* [LM6]. Entrepreneurs believe that a circular economy can promote innovation and economic development by encouraging the creation of green businesses and industries that focus on sustainable practices. For instance, the recycling industry can benefit from the collection and processing of recyclable materials, whereas companies specialising in the refurbishment of electronics and other products can thrive.

#### 4.4.2 Innovative technologies

Entrepreneurs view the implementation of innovative waste management technologies such as waste-to-energy (WtE) systems and advanced recycling facilities as essential. WtE systems convert non-recyclable waste into heat, electricity, or fuel through processes, such as combustion, gasification, and anaerobic digestion [91,92]. These technologies markedly reduce landfill waste and mitigate environmental risks, such as groundwater contamination and greenhouse gas emissions [90]. By converting waste into energy, they enhance waste management, provide alternative energy sources, contribute to energy security, and reduce fossil fuel dependence. Advanced recycling facilities that utilise cutting-edge technologies to sort, process, and recycle materials, such as plastics, metals, and electronics, are also crucial [90]. Entrepreneurs believe that these technologies are vital for achieving a circular economy in which waste is continuously repurposed and reintegrated into the production cycle, promoting environmental sustainability and economic growth.

#### 4.4.3 Community engagement and awareness

Public education campaigns are essential for deeply ingrained behaviours related to waste disposal. By implementing extensive public perception improvement strategies and educational initiatives, communities can be informed of the benefits of proper waste segregation and recycling [93]. *“So … this community engagement is more like having town hall meetings and organising events within their communities. [MS3h]. Therefore, like the community we currently work with, every last Saturday of the month, we always have cleanup within their community. So, we make it a community engagement; they have to come, we talk to them, we tell them just more about business and how they can key in.”* – [MS3]. Integrating waste management education into school curricula is another strategy to foster long-term behavioural and attitudinal change, which was found to be a critical factor in the TPB [13]. Educating teachers, children, and young adults about sustainable waste practices leads to a generational shift in attitudes towards waste management [94,95]. Additionally, public awareness campaigns in schools and community centres can involve activities such as clean-up drives, recycling competitions, and interactive sessions with waste management experts [96]. These initiatives not only raise awareness but also actively involve community members in waste management efforts, thereby increasing the efficiency of waste collection services.

#### 4.4.4 Research and development (R&D)

Through investment in R&D, Nigeria and other developing countries can discover locally appropriate solutions that consider their specific waste composition, climate conditions, and socioeconomic factors, thereby enhancing the effectiveness of MSWM strategies. *“Let us consider R&D. With this single use plastic* [holding a plastic bottle]*, we should be able to discover what we can do with them. Many things are popping out every day, so if we have a space where we have our R&D, that is really working [MS3i]. For example, instead of saying this is waste, we should encourage people to use it more because if we have what we are doing with the product, nobody is going to litter it, or another person will pick up the role of picking those things and going to make money from it. Everybody will be comfortable at the end of the day, so it is about our R&D.”* – [MS3]. Moreover, R&D informs policy-making and strategic planning by providing data-driven insights into waste generation patterns, the environmental impact of various waste management practices, and the economic viability of different waste treatment technologies [97]. Additionally, R&D fosters collaboration between academic institutions, government agencies, and the private sector and promotes a multidisciplinary approach to tackling waste management challenges [98,99].

#### 4.4.5 Public-private partnerships (PPPs)

Collaborations bring together the regulatory and enforcement powers of the government and the innovation, efficiency, and funding capabilities of the private sector [100,101]. Entrepreneurs suggest that government-only initiatives often face challenges such as limited financial resources, bureaucratic inefficiencies, and lack of technical expertise. *“This is why I believe that the government needs to collaborate with us [KM4b]. Therefore, we do not even look to the people for any form of payment or anything; we are just working with the government to carry out our own social responsibility and get* funding *from the government. It makes it easier. The government cannot be effective in reaching grassroots positions. We are at the grassroots level. Therefore, if we get money from the government, we will do this without looking at the people for a stipend.”* – [KM4]. Additionally, PPPs help address operational inefficiencies that plague many government-run waste management systems [102]. The private sector's involvement has led to better management practices, such as more regular and reliable waste collection services, improved recycling rates, and the development of sustainable waste disposal methods [103]. These partnerships also foster accountability and transparency, as private companies are often subject to performance-based contracts that incentivise efficiency and high standards of service.

#### 4.4.6 Regulatory and institutional support

Stronger regulations are seen as essential in setting clear guidelines and standards for waste disposal, recycling, and treatment [45]. Without firm enforcement of environmental laws, efforts to combat improper waste disposal and illegal dumping will continue to fall short. Entrepreneurs and community leaders alike recognise that implementing penalties for non-compliance, alongside incentives for following best practices, is key to changing behaviours. The call for dedicated, empowered, and well-funded waste management agencies resonates strongly—these institutions are viewed as critical to ensuring that policies are not just written but acted upon, creating lasting change in waste management systems. *“… But if only the government could enforce it because there are no enforcement agencies, and that is why you can still see a whole road being blocked by solid waste. [KM4c]. These are major roads, and there are people or agencies that are supposed to deal with it, and it will be there for weeks, and nobody will respond to that.”* – [KM4]. In addition to regulatory measures, strategic planning and institutional support are vital to the success of waste management initiatives. Entrepreneurs have pointed out that a lack of strategic planning has led to fragmented and inefficient waste management systems. Comprehensive plans that outline long-term goals, resource allocation, and technological adoption are necessary to create a cohesive and effective waste management framework [104]. Institutional support can facilitate collaboration between different levels of government and the private sector, fostering an environment in which innovative solutions can thrive. Bringing all these elements together will lead to more effective and sustainable waste management behaviours and practices across the country, while simultaneously creating increased opportunities for entrepreneurs within the waste management ecosystem.

## 5 Implications and limitations

Theoretically, this research expands the understanding of the role of entrepreneurs and public awareness in influencing sustainable MSWM behaviours in developing countries. Practically, the findings emphasise the importance of integrating public education with innovative business models, such as “*freemium-to-premium*” strategies, to overcome psychological barriers and enhance public engagement in MSWM. These insights suggest that targeted awareness campaigns can not only foster environmental stewardship but also drive business growth by creating demand for waste management services. Managerially, the study provides actionable recommendations for entrepreneurs and policymakers, emphasising the need for robust public-private partnerships, strategic regulatory support, and investment in research and development to address the challenges of MSWM. Overall, the findings of this article serve as compass for both municipal solid waste policymakers and entrepreneurs in their efforts to develop an effective model for increasing awareness, which can lead to sustainable waste management behaviours and foster entrepreneurship within the sector.

While this study has several strengths, a few limitations should be taken into account. First, the perspectives gathered from the seven in-depth interviews and six checklists reflected a relatively small sample size, which may potentially lead to non-representative insights. Second, the scope of the study is geographically limited to Nigeria, specifically focusing on entrepreneurs' perspectives across four geopolitical zones, including LAWMA. Although this provides valuable insights into the Nigerian context, the findings may be applied with caution in other developing countries with different socioeconomic, cultural, and regulatory environments. These limitations suggest the need for further research, including larger sample sizes, quantitative methods, and comparative studies across different regions to build a more comprehensive understanding of the role of public awareness in MSWM and entrepreneurial engagement.

## 6 Conclusion

This study brings to light the vital role that public awareness and behavioural change play in achieving sustainable waste management. Entrepreneurs working within Nigeria's MSWM sector understand that targeted education and community involvement are key to fostering responsible waste management practices. They recognise the importance of partnering with local governments, community organisations, and educational institutions to create holistic waste management plans that centre on sustainability. By tapping into innovative marketing and communication strategies, these entrepreneurs can raise awareness among their customers and the wider community about the importance of proper waste disposal. Through active stakeholder engagement and education efforts, they aim to cultivate a culture of sustainability, reducing environmental pollution while envisioning a cleaner future for Nigeria. At the same time, more efficient waste management practices offer entrepreneurs the opportunity to create jobs, boost economic growth, and enhance their business operations. This cycle of growth, supported by sustainability, not only benefits the environment but also strengthens the local economy. In this way, entrepreneurs play a pivotal role in transforming Nigeria's waste management sector—and potentially those of other developing countries—into a model of sustainability, where environmental and economic goals go hand in hand.

## Data and code availability

Data will be made available on request.

## Ethical approval

This study was approved by the Faculty of Social Science Research Ethics Committee of the University of Nottingham (FASS2023_0012/PHIR/EEE20546675).

## Funding

This study did not receive any funding.

## Declaration of Competing Interest

The authors declare that they have no known competing financial interests or personal relationships that could have appeared to influence the work reported in this paper.
