# Supplementary material for: Leveraging public awareness and behavioural change for entrepreneurial waste management
Source: Heliyon. 2024 Nov 1;10(21):e40063. doi: 10.1016/j.heliyon.2024.e40063 (PMC11566867; doi:10.1016/j.heliyon.2024.e40063)
Supplement: Multimedia component 1 [file mmc1.docx]

Appendix 1

**Interviews**

| SN | Code | Gender | Location | Region | Date and time | Duration |
| --- | --- | --- | --- | --- | --- | --- |
| 1 | CN1 | Male | Abia State | Southeast | December 15, 2023, 2:41PM | 56m 33s |
| 2 | TN2 | Male | Kaduna State | Northwest | December 13, 2023, 11:37AM | 51m 31s |
| 3 | MS3 | Male | Abuja (Airport Rd Axis) | Northcentral | December 12, 2023, 01:55PM | 58m 32s |
| 4 | KM4 | Female |  |  | December 12, 2023, 12:21PM | 30m 24s |
| 5 | CO5 | Male | Kano State (Fagge Local Government) | Northwest | December 12, 2023, 8:01AM | 45m 39s |
| 6 | LM6 | Male | Lagos | Southwest | December 11, 2023 | 1hr 26m 33s |
| 7 | AA7 | Male | Lagos | Southwest | December 14, 2023, 16:55PM | 48m 22s |

**Contributions of entrepreneurs in promoting MSWM awareness in Nigeria**

| MSWM awareness activities | Yes | No |
| --- | --- | --- |
| **High engagement activities** |  |  |
| Engaging local community leaders to communicate information regarding MSWM | 100% | 0% |
| During the COVID-19 pandemic, it seemed like almost everyone understood the importance of coming together to fight the virus. Do you think a similar strategy would be effective for creating awareness about municipal solid waste management? | 100% | 0% |
| The strictness of the penalty for waste management offences would result in greater compliance | 100% | 0% |
| **Moderate engagement activities** |  |  |
| Have individuals dedicated to actively sensitize the public on appropriate MSWM | 66.70% | 33.30% |
| Carry out frequent door-to-door outreach efforts focused on MSWM | 66.70% | 33.30% |
| Take MSWM awareness campaign to schools and educational institutions | 66.70% | 33.30% |
| **Balanced engagement activities** |  |  |
| Engaging with information hubs to address questions and concerns regarding MSWM | 50% | 50% |
| Does your organization frequently prepare posters and banners designed to convey information about municipal solid waste management | 50% | 50% |
| Organizing jingles and folk performances designed to communicate information about MSWM | 50% | 50% |
| **Low engagement activities** |  |  |
| Assisting in printing and distributing informational materials about MSWM | 33.30% | 66.70% |
| Knowledge of local networks like community associations and farmers' associations that serve as channels for disseminating information about MSWM | 33.30% | 66.70% |
| Engage town criers and community radio to create awareness of MSWM | 33.30% | 66.70% |
| Having a dedicated toll-free helpline established to provide pre-recorded messages related to MSWM | 16.70% | 83.30% |
| Have mobile applications or SMS-based services that are designed to provide updates or share information related to MSWM | 0% | 100% |
